# Place of upbringing in early childhood as related to inflammatory bowel diseases in adulthood: a population-based cohort study in Northern Europe

**DOI:** 10.1007/s10654-014-9922-3

**Published:** 2014-06-11

**Authors:** Signe Timm, Cecilie Svanes, Christer Janson, Torben Sigsgaard, Ane Johannessen, Thorarinn Gislason, Rain Jogi, Ernst Omenaas, Bertil Forsberg, Kjell Torén, Mathias Holm, Lennart Bråbäck, Vivi Schlünssen

**Affiliations:** 1 Department of Public Health, Aarhus University, Bartholins Allé 2, Building 1260, 8000 Århus, Denmark; 2 Institute of Clinical Science, University of Bergen, Bergen, Norway; 3 Department of Medical Sciences, Respiratory Medicine and Allergology, Uppsala University, Uppsala, Sweden; 4 Centre for Clinical Research, Haukeland University Hospital, Bergen, Norway; 5 Department of Respiratory Medicine and Sleep, Landspitali - The National University Hospital of Iceland, Reykjavík, Iceland; 6 Faculty of Medicine, University of Iceland, Reykjavík, Iceland; 7 Lung Clinic, Tartu University Hospital, Tartu, Estonia; 8 Department of Public Health and Clinical Medicine, Umeå University, Umeå, Sweden; 9 Section of Occupational and Environmental Medicine, University of Gothenburg, Göteborg, Sweden; 10 Occupational Medicine, Respiratory Diseases and Toxicology, University of Perugia, Perugia, Italy; 11 Department of Occupational and Environmental Medicine, University of Gothenburg, Göteborg, Sweden; 12 Department of Occupational Medicine, Danish Ramazzini Centre, Aarhus University Hospital, Århus, Denmark

**Keywords:** Inflammatory bowel disease, Ulcerative colitis, Crohn’s disease, Microbial exposure, Rural/urban environments, Hygiene hypothesis

## Abstract

*Background* The two inflammatory bowel diseases (IBD), ulcerative colitis and Crohn's disease, has increased rapidly during the twentieth century, but the aetiology is still poorly understood. Impaired immunological competence due to decreasing biodiversity and altered microbial stimulation is a suggested explanation. *Objective* Place of upbringing was used as a proxy for the level and diversity of microbial stimulation to investigate the effects on the prevalence of IBD in adulthood. *Methods* Respiratory Health in Northern Europe (RHINE) III is a postal follow-up questionnaire of the European Community Respiratory Health Survey (ECRHS) cohorts established in 1989–1992. The study population was 10,864 subjects born 1945–1971 in Denmark, Norway, Sweden, Iceland and Estonia, who responded to questionnaires in 2000–2002 and 2010–2012. Data were analysed in logistic and Cox regression models taking age, sex, smoking and body mass index into consideration. *Results* Being born and raised on a livestock farm the first 5 years of life was associated with a lower risk of IBD compared to city living in logistic (OR 0.54, 95 % CI 0.31; 0.94) and Cox regression models (HR 0.55, 95 % CI 0.31; 0.98). Random-effect meta-analysis did not identify geographical difference in this association. Furthermore, there was a significant trend comparing livestock farm living, village and city living (*p* < 0.01). Sub-analyses showed that the protective effect was only present among subjects born after 1952 (OR 0.25, 95 % CI 0.11; 0.61). *Conclusion* This study suggests a protective effect from livestock farm living in early childhood on the occurrence of IBD in adulthood, however only among subjects born after 1952. We speculate that lower microbial diversity is an explanation for the findings.

## Introduction

A rapid increase in prevalence of the two inflammatory bowel diseases (IBD), ulcerative colitis and Crohn’s disease, has been observed during the second half of the twentieth century [1, 2]. A broad range of potential risk factors have been investigated, mainly in case–control studies, but the aetiology is still poorly understood [2, 3]. Although genetic risk factors for both diseases have been identified, they can not account for the rapidly increasing prevalence alone and the differences in incidence and prevalence across time, geographic regions and populations suggest that environmental factors play an important role in the aetiology of IBD [2–5].

The microbial environment of mankind has undergone massive changes throughout the human evolution, and the prevalence of IBD has increased in continuation of industrialization and urbanisation during the second half of the twentieth century [6, 7] These time trends—including changes in hygienic standards, family circumstances, dietary habits and living conditions—have changed the microbial environment and seem to contribute to the modern “Westernized” picture with high incidence of immunoregulatory disorders such as IBD [4, 8–13]. Several studies suggest that the “hygiene hypothesis”, proposing impaired immunological competence with low microbial stimulation in early childhood, may be relevant not only to asthma and allergy, but also to IBD [3, 6, 14–19]. The early work with the hygiene hypothesis was dominated by the conviction that Th1 and Th2 lymphocytes were mutually antagonistic, and that the decreased microbial exposure led to a reduced Th1 activity. This was thought to result in a compensatory Th2 dominated immune system and posed the explanation for the rising incidence of Th2-mediated allergies. Although it seems biologically plausible, there is a lack of evidence to support this view at disease development, and the model is insufficient to explain why Th1-mediated diseases as Crohn’s disease is increasing in parallel. Further investigation in both human and animal studies suggests that the Th1/Th2 paradigm might be an over-simplification, and that other immunoregulatory cells, for instance regulatory T cells, also play a role [6, 15, 16, 20–24]. Both murine and human studies on IBD indicate that the mucosal immune function can resolve itself into dominant activity of specific T cell effector pathways, which is an important implication for the understanding of the mechanisms [23].

Children who are growing up on a farm are exposed to a wider range of microbes than their counterparts in the cities [25]. Because IBD occurs mainly in developed urbanised countries, and steadily rising in developing countries like India and China as they become more urbanised, the deprivation of microbial exposure measured by a decline in rural exposure is speculated to have an impact [26, 27]. An immigration study from British Columbia suggests that compared to the natives, South Asian pediatric 2nd generation immigrants not only adapt the incidence rate in the new country, they actually show higher incidence rates of IBD [28]. In another study, this was not the case for the adult immigrants, suggesting that age at the time of migration is crucial [29].

There is a need for more studies to understand how modern lifestyle may influence the risk of IBD and microbial exposure in early life is of great interest to further research [1–3, 5, 12]. The aim of this study was therefore to investigate the role of place of upbringing in early childhood on the occurrence of IBD in adulthood.

## Materials and methods

### Study population

The original study population was >150,000 randomly selected men and women born between 1945 and 1973, who participated in stage 1 of The European Community Respiratory Health Survey (ECRHS) during 1989–1992 [30]. Each of the 48 participating centres recruited at least 1,500 men and 1,500 women aged 20–44 years.

**Fig. 1:**
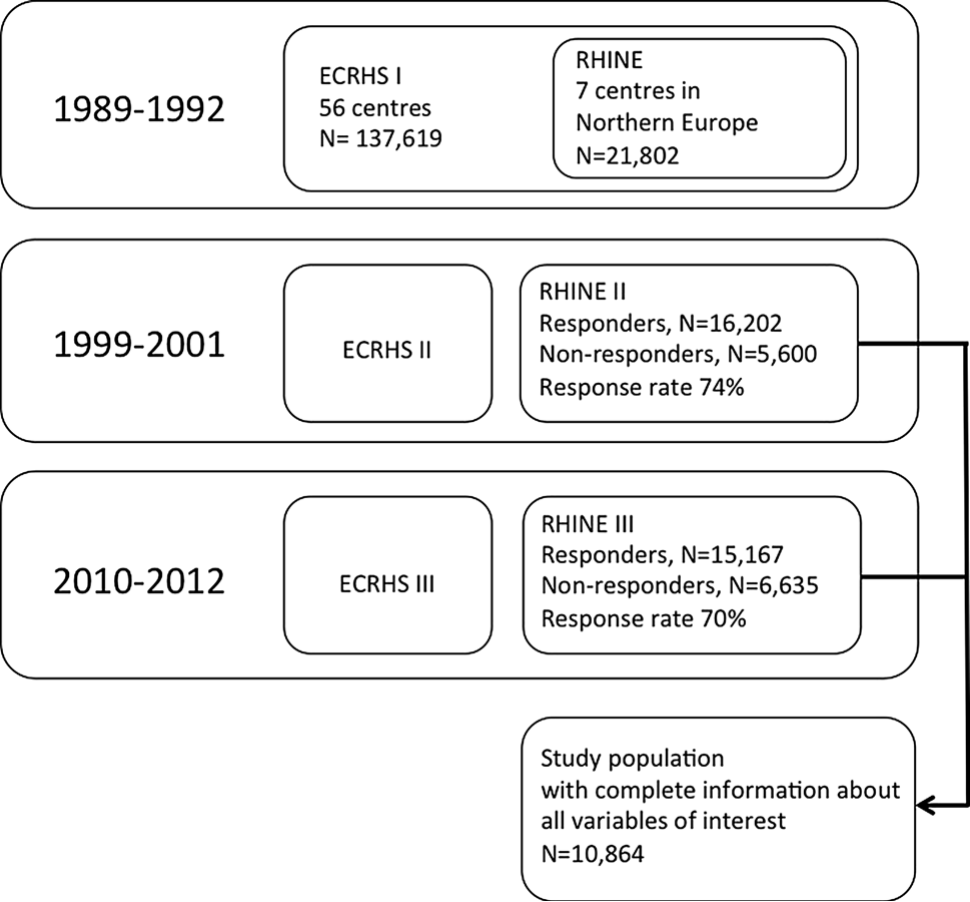
Flow chart for the times of follow-up and identification of the study population

The study population RHINE (Respiratory Health in Northern Europe) consisted of the 21,802 men and women from the ECRHS population in the seven centres located in northern Europe—Reykjavik in Iceland; Bergen in Norway; Umeaa, Uppsala and Gothenburg in Sweden; Tartu in Estonia; and Aarhus in Denmark. In 1999–2001 all RHINE subjects were sent a postal questionnaire (RHINE II), which was answered by 16,202 subjects (74 %). At follow-up in 2010–2012 15,167 subjects (70 %) responded to the RHINE III questionnaire [31, 32]. A flow chart of the study population is shown in Fig. 1.

### Variables and data measurement

A formal forward/backward translation of the questionnaires was carried out to ensure validity. Questions regarding IBD and place of upbringing were asked in the RHINE III questionnaire, and place of upbringing was considered as a proxy for the level and diversity of microbial exposure in early childhood. IBD was defined as a positive answer to either “Do you have or have you ever had ulcerative colitis?” or “Do you have or have you ever had Crohn’s disease?” and a reported year of onset due to the answer of “How old were you when the disease started?”. Place of upbringing was defined due to the answer of “What term best describes the place you lived most of the time when you were under the age of 5 years?” with response categories (1) farm with livestock, (2) farm without livestock, (3) village in rural area, (4) small town, (5) suburb of city and (6) inner city. Response 1 was analysed as “livestock farm”, response 2–5 were merged and analysed as “village”, and response 6 was analysed as “city”.

The confounder variables were selected a priory on the basis of existing knowledge of possible risk factors and modifying factors in IBD and were collected from the RHINE II questionnaire. These were age, sex, smoking and body mass index (BMI). Information on smoking was collected due to the answers of the questions “are you a smoker? (this applies even if you only smoke that odd cigarette/cigar or pipe every week)” and “are you an ex-smoker?”. BMI was calculated from self-reports of height and weight from the questions “How tall are you?” and “How much do you weigh?”.

### Statistical methods

Statistics were calculated using STATA 12.1 (STATA Corp, College Station, Texas, USA). The analyses were conducted on subjects with complete information on all variables included in the models. Data were analysed in both logistic and Cox regression models adjusting for age, sex, smoking and BMI, and presented by odds ratios (OR) and hazard ratios (HR) with corresponding 95 % confidence intervals (95 % CI), respectively. The reference was set at female and never-smoker.

Furthermore a random-effects meta-analysis was performed to explore heterogeneity between centres. Additional analyses included test for trend, estimation of incidence and incidence rate ratios, and logistic regression stratified by year of birth. Due to the low numbers of IBD-cases among young subjects, who had lived on a livestock farm (2 cases born after 1958), the stratification was conducted for subjects above and below the upper age-quartile (birth year 1952).

## Results

Basic characteristics for the study population (N = 10,864) are shown in Table 1. The population contained a total of 179 IBD-cases (1.65 %): 49 cases with Crohn’s disease (0.45 %), 140 cases with ulcerative colitis (1.29 %) and 10 cases with both diseases (0.09 %). This corresponds to an incidence of 31 per 100,000 person years. The number of cases according to place of upbringing was 19, 121 and 39 for livestock farm, village and city respectively. Subjects in the respective exposure groups were comparable regarding age and BMI. Subjects who grew up in a city were more likely to be current smokers, and subjects who grew up on a livestock farm were more likely to be women and never-smokers.

**Table 1:** Characteristics of the study population

	Livestock farm	Village	City	All
Subjects (N)	1.496	7.677	1.691	10.864
Cases [N (%)]	19 (1.27 %)	121 (1.58 %)	39 (2.31 %)	179 (1.65 %)
Age in 2011 (mean ± SD)	55.8 ± 6.6	52.5 ± 7.1	53.6 ± 7.0	53.1 ± 7.1
Sex [N (% F)]	858 (57.3 %)	4,161 (54.2 %)	861 (50.9 %)	5,880 (54.1 %)
BMI (mean ± SD)	24.9 ± 4.2	24.5 ± 3.9	24.8 ± 4.2	24.6 ± 4.0
Smoking status				
Current smoker [N (%)]	366 (24.5 %)	1,925 (25.1 %)	543 (32.1 %)	2,834 (26.1 %)
Ex-smoker [N (%)]	373 (24.9 %)	2,053 (26.7 %)	443 (26.2 %)	2,869 (26.4 %)
Never smokers [N (%)]	757 (50.6 %)	3,699 (48.2 %)	705 (41.7 %)	5,161 (47.5 %)

**Fig. 2:**
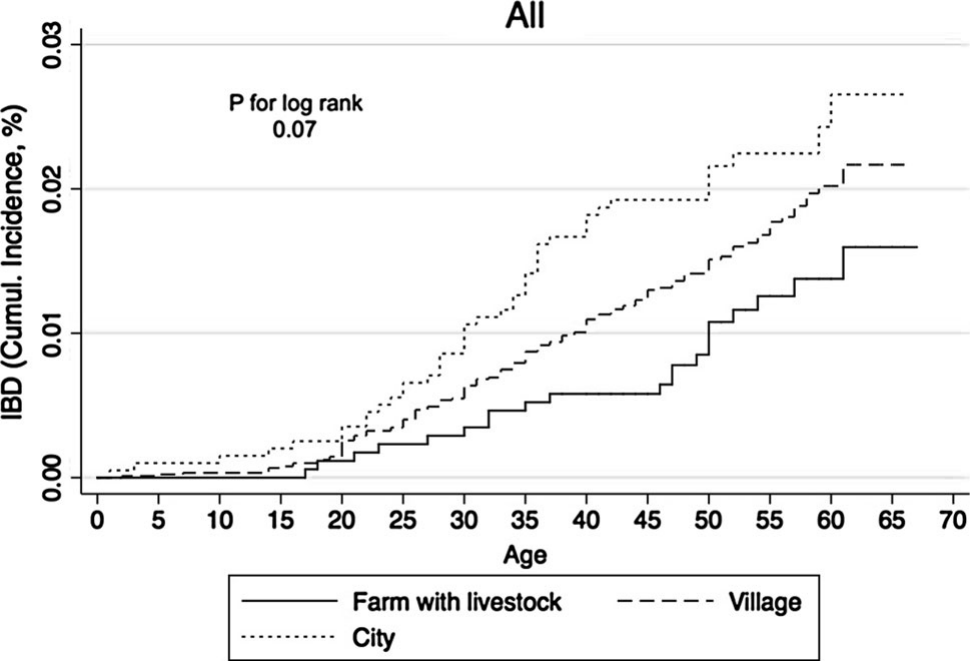
Kaplan–Meier curve plotting cumulative incidence of IBD dependent on place of upbringing

In multiple logistic regression analysis, living on a livestock farm the first 5 years of life was significantly associated with a lower risk of IBD compared to city living (OR 0.54, 95 % CI 0.31–0.94), Table 2. The multiple Cox regression analysis showed similar results (HR 0.55, 95 % CI 0.31–0.98). The models were extended to include centre, and this showed similar results. Test for trend comparing livestock farm living, village living and city living was found statistically significant in both models (*p* < 0.05). This trend was further supported by the Kaplan–Meier curve plotting the cumulative incidence as a function of age (Fig. 2).

**Table 2:** Multiple logistic regression and Cox regression models of place of upbringing and IBD adjusting for age, sex, smoking and BMI

	Logistic regression	Cox regression
	OR (95 % CI)	HR (95 % CI)
Place of upbringing		
Livestock farm	0.54 (0.31–0.94)	0.55 (0.31–0.98)
Village	0.71 (0.49–1.03)	0.75 (0.52–1.10)
City	1	1
*P* for trend	0.02	0.04

**Fig. 3:**
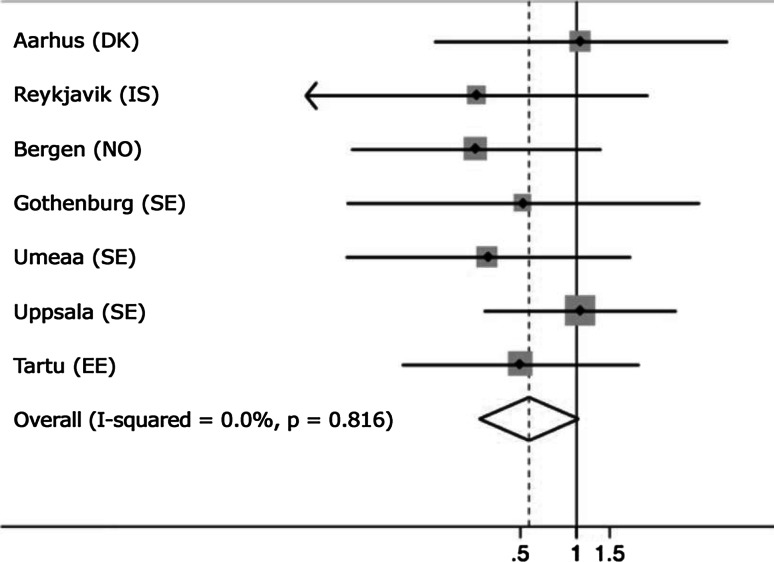
Forrest plot with OR (*dots*) and 95 % CI (*lines*) showing the effect of livestock farm living compared to city living across participating centres. Results are adjusted for age, sex, smoking and BMI. The *diamond* indicates 95 % CI for the combined OR from model with random effects. The size of each *square* is proportional to the sample size. The overall prevalence was 1.65 %, and the centre specific prevalence is as follows: Aarhus (DK) 1.45 %, Reykjavik (IS) 2.13 %, Bergen (NO) 2.17 %, Gothenburg (SE) 1.63 %, Umeaa (SE) 0.84 %, Uppsala (SE) 1.82 %, Tartu (EE) 1.48 %

The random-effect meta-analysis showed no significant heterogeneity between centres (*p* = 0.82, I^2^ = 0 %, Fig. 3).

Sub-analyses stratified by year of birth revealed that the protective effect of livestock farm living was only present among subjects born after 1952, Table 3. A test for trend was significant among subjects born after 1952 (*p* < 0.01), but not among subjects born in 1952 or before (*p* = 0.34).

**Table 3:** Multiple logistic regression models of place of upbringing and IBD stratified by year of birth and adjusted for sex, smoking and BMI

	Born after 1952	Born 1952 or before
	OR (95 % CI)	OR (95 % CI)
Place of upbringing		
Livestock farm	0.25 (0.11–0.61)	1.64 (0.64–4.20)
Village	0.52 (0.34–0.79)	1.60 (0.71–3.60)
City	1	1
*P* for trend	<0.01	0.34
N (cases)	7,785 (118)	3,079 (61)

**Fig. 4:**
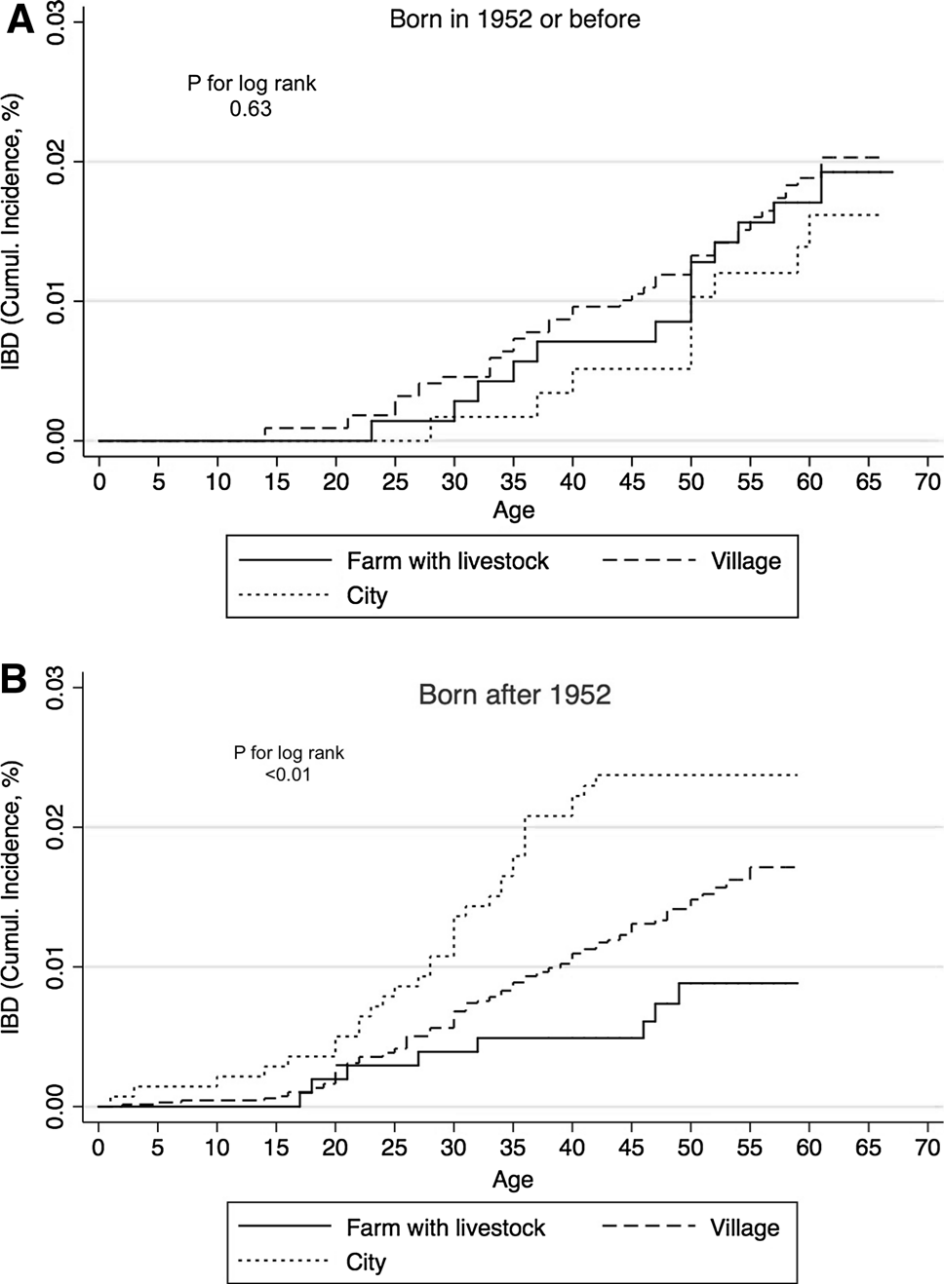
Kaplan–Meier curves plotting cumulative incidence as a function of age for the two strata **a** born in or before 1952 and **b** born after 1952. The number of cases in each strata was 61 and 118, respectively

The stratified Kaplan–Meier curves (Fig. 4a, b) further revealed that growing up in a city, was associated with the highest incidence of IBD among subjects born after 1952 and the lowest incidence of IBD among the subjects born in 1952 or before; however, this difference did not reach statistical significance, incidence rate ratio 1.92 (95 % CI 0.92–4.55).

## Discussion

In this population-based cohort study, subjects who lived their first 5 years of life on a livestock farm had significantly less IBD in adulthood as compared to those who grew up in a city or village. This finding was consistent by different analytical approaches, and appeared to be consistent across geographical regions including Estonia and other northern European regions. Stratifying by year of birth showed that the protective effect of livestock farm living was convincing only among subjects born after 1952.

The population based follow-up design is an important strength of this study even though questions about exposure and outcome are asked in the same questionnaire. The true place of upbringing does not change over time, and we therefore assess that this is a methodological and not practical issue. The originally six rural–urban categories were merged into three, namely the two extremes (livestock and city) and the four in between due to the interest in studying the contrast between livestock farm and city. Post hoc analysis showed no trend in the prevalence of IBD in the 4 merged groups (farm without livestock, village in rural area, small town and suburb of city).

To our knowledge, this study is one of the first to investigate IBD and place of upbringing in survival models [12]. A further strength is that the studied countries are among the IBD high-incidence countries in Europe and therefore represent a relevant geographical area to study for clues to the emergence of IBD [26, 33]. The incidence was similar to a Swedish population-based study from 2005 to 2009 and slightly higher compared to a Danish population-based study from 2002 based on hospital-admissions in a specific county in Denmark [34–36]. A recent European multi-centre study from 2013 reported similar incidence rates for Denmark and Iceland, and remarkably lower rates for Estonia [37].

All variables of interest are self-reported and a limitation is therefore the potential risk of recall bias. The real place of upbringing does not change over time, and we expect subjects to answer truthfully and to be able to remember this information. Furthermore the possible association between place of upbringing and health is not commonly known, and we therefore assess the bias due to recall being minimal. Regarding information on IBD, it is likely that some patients may have mistaken the inflammatory bowel diseases with for instance irritable bowel syndrome. We have no information on clinical diagnosis to investigate this, but on the other hand we do not expect the potential misclassification to be correlated with exposure in early life, and we therefore expect the potential misclassification to be non-differential. Despite the relative large study population, another limitation is the lack of power. The proportion of cases was low compared to non-cases contributing to limited power when analysing sub-groups. Furthermore, we cannot distinguish between ulcerative colitis and Crohn’s disease. The two diseases have clinical and immunological differences and the risk factors differ at least to some extent. Smoking is for instance increasing the risk of Crohn’s disease but may actually have a protective effect on ulcerative colitis, which we have also found in our study [38]. Despite the limited power in disease specific analyses, we have nevertheless done explorative analyses on incidence rates due to place of upbringing and found the same patterns as for the merged analyses. This was only significant for ulcerative colitis, though (data not shown).

The impact of selection bias is difficult to assess because each centre could influence the recruitment strategy most likely to maximize response [39]. The default was to make a random sample, but how this random sample was selected may have varied between countries according to the variation in registers available for randomization. The majority of the studied countries have a public health system with free and equal access to health care, but it is possible that the geographic distance to health care varies between the countries. We do not believe that the proneness to seek health care differs between people who grew up on a livestock farm and in a village, but it is likely that those who grew up in a city are more inclined to seek health care with light symptoms than their rural counterparts.

An analysis of loss to follow-up in the RHINE population suggests a minimal non-significant difference in associations between respiratory symptoms and sex and age among responders and non-responders during follow-up [40]. However, this study does not specifically take non-participation and IBD-status or associations between IBD and place of upbringing into account, but we have no reason to believe that the association between upbringing and IBD is affected by selection bias. Unfortunately, the study was not originally designed to investigate IBD, and we have therefore no information about the IBD-status before 2010 when the RHINE III questionnaire was completed.

A methodological limitation is the retrospective procedure of estimates for risk and occurrence. These estimates require that subjects were alive and not emigrated or out of reach at follow-up in 2010–2012, and were able to retrospectively report the time of onset for IBD. In addition, the rapidly increased prevalence and following attention of IBD may have influenced physicians to become more aware of the disease and diagnosis, and could potentially mean that the interval between disease onset and time of diagnosis vary in the period. The confounder variables were assessed in 1999–2001 when some of the cases have already got the diagnosis and some have not yet. The adjustment may be problematic if the majority of the cases suffered from IBD i.e. before they started smoking. We investigated that in our data and found that only nine cases started smoking after the time of disease onset, and we therefore found it defensible to keep the model as it is. Furthermore, we have investigated the model before and after inclusion of educational status and found no difference between the estimates. Since the literature on the impact from socioeconomic status in IBD is inconsistent, we therefore omitted the variable from the model [41, 42].

The Cox regression models assumes the subjects to be at risk their whole life, even though the marker of microbial exposure is only valid for the first 5 years of life. Recent studies suggest that early life exposure may provide lifelong effects on the immunoregulatory properties, but it may still be questioned whether this is a reasonable assumption or not [16, 43]. Because of these conditions the estimates will bear a mark of “pseudo-estimates”, and must be interpreted with this limitation in mind.

The findings from this study are overall in line with the current evidence. Three case–control studies have revealed farm living as a protective factor against Crohn’s disease, but not against ulcerative colitis [1, 44, 45]. Notably, the timing of farm exposure varied between the studies. Gearry et al. [1] investigated three time windows—infancy, childhood and adulthood—and found the association for childhood and adulthood only. Wurzelmann et al. [44] investigated three time windows according to age—0–5, 6–11 and 12–15 years—and found a significant trend association of risk increasing with three levels of urban living relative to farm living among the 0–5 years old. Bernstein et al. [45] did not investigate a specific time window, while they found a protective association for subjects, who had lived on a farm at any time in their lives. López-Serrano et al. and Feeney et al. [41, 46] found similar tendencies for both Crohn’s disease and ulcerative colitis, but the estimates were not significant in the latter. A systematic review and meta-analysis by Soon et al. [12] found a positive association between urban environment and the occurrence of both ulcerative colitis and Crohn’s disease, but no consistent time window was identified. The results is similar to the findings from a register-based Canadian study conducted by Green et al. [47], and findings from a cross-sectional population-based Israeli study conducted by Klement et al. [48]. However, a recent case–control study on >500 patients with ulcerative colitis or Crohn’s disease did not find a significant association with urban upbringing. The different results may be due to different study designs, but most importantly their exposure variable (urban vs. rural) differs from the one used in our study where upbringing on farms with animals seems to be crucial for the protective effect [42].

Our findings are moreover comparable to those from a population-based incidence study in Sweden. Ekbom et al. [49] found that urban areas had higher age-adjusted incidence rates than rural areas for both Crohn’s disease and ulcerative colitis. Ekbom et al. also suggests that subjects born after 1945 had higher incidence rates of both Crohn’s disease and ulcerative colitis and we confirm this for the urban population, where the highest incidence rate of IBD was found among subjects born after 1952.

Changing microbial environments following changing characteristics of urban and rural living may contribute to explain our findings. Living in an urban setting is associated with a predominance of several significant risk factors for IBD including more smoking and antibiotic use, and less helminths exposure [12]. In addition, a recent study suggests an association between air-pollution and IBD in urban settings [50]. Living in rural settings is in contrast associated with several significant protective factors including increased exposure to non-pathogen immunoregulatory microorganisms [6, 8, 51], markers of dietary habits such as having a vegetable garden during childhood [1] as well as a high frequency of drinking unpasteurized milk as a child [45]. Having been breastfed at least the first 3 months of life was also found to reduce the risk of both Crohn’s disease and ulcerative colitis in a case–control study, and to reduce the risk of IBD in general in a systematic review [1, 52]. The other way around, it has been suggested that “urban diet”, which contains large quantities of inert inorganic non-nutrient microparticles (for instance food additives), may be an explanation for the rising incidence of Crohn’s disease in urban settings [53]. Additionally, contact with farm animals (cattle, pigs, sheep or goats) during the first year of life have been demonstrated to reduce the risk of both Crohn’s disease and ulcerative colitis by half among juvenile subjects [54]. Current evidence for respiratory allergies also suggests, that the protective effect of farm living is associated mainly with livestock exposure, because the animals mediate the microbial exposure in these environments, but the exact mechanisms behind this perception is still debated [54, 55].

There seems to be clear similarities in the association between microbial exposure and IBD and asthma/allergic diseases. There is an emerging body of evidence supporting an important role of the microbial environment in allergic diseases including asthma. Studies have shown that farm upbringing and farm work reduces the risk of allergy, and for asthma, a reduction has now been explained by exposure to greater microbial diversity [25, 56–60]. Bråbäck et al. assessed the trends of asthma and allergic diseases among Swedish conscripts over a period of three decades. An increasing prevalence of asthma was only observed in cohorts born after 1960 and a protective effect of farm living on asthma was only observed in cohorts born after 1970. In contrast, allergic rhinitis and eczema showed a continuous increase over the whole study period, but the protective effect of farm living was greater in later cohorts [61].

There can be several explanations for the differences between birth cohorts. First, it is feasible that the differences in microbial exposure between urban and rural settings have increased over time and one may suspect poor biodiversity in modern urban settings. The birth year of the subjects might further be a proxy also for other early life exposures.

## Conclusion

In conclusion, this study suggests a protective effect from livestock farm living in early childhood on the occurrence of inflammatory bowel diseases in adulthood in a northern European population. This finding was only detected among younger birth cohorts, for whom a modern urban environment may reflect lower biodiversity than for older birth cohorts. Further, the findings appeared to be consistent between regions, including Western and former Eastern European centres, and with some differences in farming traditions. These findings support the hypothesis that low biodiversity and related changes in microbial exposure might be of importance in the aetiology of inflammatory bowel diseases. Moreover, the observations strongly suggest a role for early life environment for development of inflammatory bowel diseases, as is previously shown for a range of other chronic diseases.
